# IL-17A Mediates Demyelination by Activating A1 Astrocytes *via* SOCS3 During *Angiostrongylus cantonensis* Infection

**DOI:** 10.3389/fimmu.2022.845011

**Published:** 2022-02-28

**Authors:** Zongpu Zhou, Tuo Lin, Zhen Liu, Qian Ding, Zhixuan Ma, Wanqi Li, Fukang Xie, Yue Lan, Ying Feng

**Affiliations:** ^1^ School of Medicine, South China University of Technology, Guangzhou, China; ^2^ Department of Rehabilitation Medicine, Guangzhou First People’s Hospital, School of Medicine, South China University of Technology, Guangzhou, China; ^3^ Department of Blood Transfusion, Guangzhou First People’s Hospital, Guangzhou, China; ^4^ Zhongshan School of Medicine, Sun Yat-Sen University, Guangzhou, China

**Keywords:** *Angiostrongylus cantonensis*, IL-17A, demyelination, astrocyte, SOCS3, IL-17RA/SOCS3/STAT3 pathway

## Abstract

**Background:**

Demyelinating disease of the central nervous system is one of the most common neurological diseases and effective treatment is still under in-depth research. Our previous study showed that *Angiostrongylus cantonensis* infection can induce demyelination injury in mouse brains and IL-17A expression was shown to be significantly increased during this process. Moreover, we found that IL-17A inhibition attenuated the demyelination caused by *A. cantonensis* infection. However, the underlying mechanisms have not yet been fully elucidated.

**Methods:**

IL-17A neutralizing antibodies were injected into *A. cantonensis* infected mice to decrease IL-17A levels. The activation of glial cells in the brain and the expression of cell markers were detected by a variety of methods, including real-time quantitative PCR, western blotting, and immunofluorescence staining. The relationship between IL-17A and astrocyte activation was further identified by *in vitro* experiments. The role of SOCS3 in the IL-17A stimulating process was determined using RNA-seq data collection of infected mice and the siRNA interference method.

**Results:**

Demyelination of the corpus callosum was relieved after administration of IL-17A neutralizing antibody and this was accompanied by decreased activation of A1 type astrocytes around this region. The expression of SOCS3 was attenuated and activation of astrocytes by IL-17A was mediated by the IL-17RA/STAT3/SOCS3 pathway. IL-17A not only directly damaged oligodendrocytes but also indirectly damaged oligodendrocytes through A1 astrocyte mediation. Specific siRNA inhibition of IL-17A-inducible SOCS3 in astrocytes alleviated their damaging effects on oligodendrocytes.

**Conclusion:**

IL-17A plays an important role in demyelination induced by *A. cantonensis* infection *via* the IL-17RA/STAT3/SOCS3 pathway in A1-type astrocytes, indicating that specific blockage of IL-17A and SOCS3 activity could be a therapeutic strategy for neuroinflammatory demyelinating diseases associated with astrocyte activation.

## Introduction

Angiostrongyliasis is a food-borne parasitic disease caused by infection with *Angiostrongylus cantonensis*, a parasite found mostly in coastal areas. In recent years, outbreaks of this disease have been reported, such as the famous “Ampullaria gigas infection” incident in 2006 (1). Humans are mainly infected by eating raw snails infected with third-stage larvae of *A. cantonensi*s or contaminated vegetables and fruits (2). The clinical manifestations of infection are headache, neck stiffness, vomiting, and fever and the pathological changes are mainly meningitis and meningoencephalitis characterized by increased eosinophils (3). After antiparasitic therapy combined with hormone therapy, patients can generally be cured; however, most patients continue to have symptoms such as skin paresthesia, limb weakness and decreased vision, which seriously affects the quality of life of these patients. If antiparasitic drugs are used alone in the late stage of infection, the disintegration products released after the death of *A. cantonensis* can induce a secondary severe inflammatory response in the brain tissue, thus aggravating the damage to the brain tissue (4). Therefore, investigating the pathological mechanism of *A. cantonensi*s infection and optimizing the treatment is still a focus for researchers.

Neuronal apoptosis is related to abnormal motor behavior and a study showed a large number of apoptotic neurons were detected in the cortical and hippocampal regions of rats and mice infected with *A. cantonensis* (5). Furthermore, significant activation of microglia in the brain was observed after infection and increased levels of various microglia-associated inflammatory factors were also detected. It has been shown that microglial activation can be induced *via* stimulation by *A. cantonensis* antigens *in vitro* (6). The above evidence indicates that, in addition to typical eosinophilic meningitis, *A. cantonensi*s infection can also lead to a variety of other brain parenchymal lesions. It has been reported that patients with *A. cantonensis* infection were misdiagnosed with multiple sclerosis because of several similar symptoms, including headaches, limb paresthesia, and urinary retention (7). Furthermore, the MRI results for these patients showed spot-like lesions in the subcortical frontal lobe and non-enhancement lesions in two cervical vertebrae, indicative of multiple sclerosis; however, *A. cantonensis-associated* antigens were detected in the patient’s cerebrospinal fluid. Demyelinating lesions on the sagittal surface of the brain have been detected in mice infected with *A. cantonensi*s and several myelin-related indicators have also been shown to increase in the brain lavage fluid (8). A previous study by our group also showed that *A. cantonensi*s infection can lead to demyelination of the mouse brain and optic nerve. MRI examination of the mouse brain showed that the corpus callosum area was highlighted and electron microscopy and LFB staining showed demyelination lesions. Visual impairment and demyelination changes to the optic nerve have also been observed in mice (9). Central nerve demyelinating diseases mainly include two types: myelin formation disorder and myelin destruction (10). The former is caused by congenital dysplasia or genetic mutations, such as leukodystrophy, while the latter refers to demyelination due to inflammation caused by infection, autoimmune response, nutrition deficiency, or drug-induced injury (11). To date, the pathogenesis of the demyelinating disease is not fully understood; hence, current treatments and their efficacy are very limited.

Astrocytes are the most abundant glial cells in the brain. They are widely distributed in various regions of the brain, participate in the formation of the blood-brain barrier (BBB), maintain homeostasis in the brain, and are responsible for the immune monitoring and cell support functions of the brain (12, 13). In recent years, astrocytes have been roughly divided into A1 and A2 phenotypes according to gene expression and cell function, where A1 astrocytes promote inflammation and A2 astrocytes inhibit inflammation (14). The expression of the Interleukin-17 (IL-17) receptor can be detected on the surface of astrocytes in both human and mouse brain tissue and astrocytes can also secrete IL-17 after stimulation by inflammation (15). IL-17 inhibits the differentiation of neural stem cells into astrocytes during neural development (16). In a cerebral ischemia injury model, IL-17 and TNF-α can synergistically promote astrocyte secretion of CXCL1, an inflammatory chemokine of neutrophils, and can aggravate the severity of inflammatory response in the ischemic regions (17). In the pathogenesis of multiple sclerosis, the number of IL-17 receptors on the surface of astrocytes has been shown to increase. IL-17 stimulates astrocytes located on the BBB, causing changes to the permeability of the barrier and aggravating the progression of the disease (18).

Suppressors of cytokine signaling (SOCS) proteins are involved in the regulation of gene expression and are mainly expressed in immune cells and nerve cells (19). SOCS proteins can act on the JAK/STAT pathway by regulating the phosphorylation of STAT proteins (20). SOCS3 protein, a member of the SOCS family, inhibits the expression of many genes. In experimental autoimmune encephalomyelitis (EAE) models, miR-409-3p and miR-1896 are involved in the production of IL-17-induced inflammatory cytokines secreted by astrocytes because they target the SOCS3/STAT3 signaling pathway (21). Knockout of SOCS3 can promote the synthesis of Th17 cells because SOCS3 can regulate the synthesis of IL-23, which is also a key cytokine for Th17 formation (22). The function of SOCS3 in neurological diseases has also been studied because its expression has been detected in a variety of cells in the brain, including astrocytes, microglia, oligodendrocytes, and neurons (23), indicating that the SOCS3 gene is closely related to the development and damage of brain tissues. In a spinal cord injury model, conditional knockout of the SOCS3 gene in brain cells expressing nestin can inhibit infiltration of inflammatory cells and apoptosis of neurons and oligodendrocytes (24).

This study was based on our previous research on the role of glial cells in *A. cantonensis* induced brain damage (25). We found that IL-17A activated numerous astrocytes and this may be an important cause of IL-17A-mediated demyelination injury. To further confirm this hypothesis, we used a medium transfer and co-culture system to test the effect of IL-17A activated astrocytes on oligodendrocytes. In addition, the expression level of IL-17A was positively correlated with the SOCS3 during *A. cantonensis* infection. SOCS3 siRNA was applied in astrocyte medium to inhibit SOCS3 expression and we verified that IL-17A stimulates astrocytes through IL-17RA, STAT3, and SOCS3. The results showed that IL-17A can activate A1 astrocytes by upregulating SOCS3 expression level, which in turn damages oligodendrocytes. We hope to shed new light on the functions of IL-17A in brain inflammatory injury, to highlight the need for further revealing the pathogenesis of the demyelinating disease, allowing for optimization of existing treatment plans and proposing new treatment methods.

## Methods

### Establishment of *A. cantonensis* Infection Model and Anti-IL-17A Antibody Treatment

Male BALB/c mice were purchased from the Animal Center Laboratory of Sun Yat-Sen University (Guangzhou, China). The Institutional Animal Care and Use Committee approved all animal procedures. All mice were raised in the same room and were randomly divided into experimental and control groups. We collected larvae III (L3) of *A. cantonensis* from Biomphalaria glabrata and washed them from the snail sediment with phosphate-buffered saline (PBS). Larvae number was counted using an anatomical microscope. Gavage administration method was applied to inject 30 A*. cantonensis* L3 into experimental group mice stomach. IL-17A neutralizing antibody (0.05 ×10^-3^mg/kg/day, eBioscience) or immunoglobulin G1 (IgG1) isotype control (clone MOPC-21) were separately administrated to the experimental group and control group for 3 weeks through intraperitoneal injection method. We started antibody injection from 3 days before *A. cantonensis* infection to avoid off-target effects of antibiotics.

### Astrocyte Isolation, Culture and SOCS3 siRNA Interference

Neonatal mice (1-3 days) old were selected and decapitated under aseptic conditions. The brains were placed in cold HBSS solution, and the meninges and blood vessels were dissected with instruments under an anatomical microscope. Then the tissue was cut and transferred to trypsin digestion solution (containing 0.1%DNA enzyme). Tissues were digested at 37°C for 20-30min. After digestion, DMEM10S medium (DMEM/F12 supplemented with 10% FBS) was added to terminate digestion. After centrifugation, the supernatant was removed and re-added to the medium to suspend precipitation. The cells were transferred into a poly-d-Lysine (PDL) coated culture flask and cultured for 7-10 days after mixed culture. The culture flask was placed on a constant temperature rotary shaker at 37°C and 250 RPM for 1 h to wipe off microglia and was continuously placed on the rotary shaker for 17-18h to isolate oligodendrocytes by differential adhesion. Primary astrocytes were seeded in 6-well plates and transiently transfected using riboFECT™ CP Reagent. Cells received serial murine SOCS3 deletion mutants and different transfected concentrations to verify the best interfering sequence and concentration. We used fluorescence labeling siRNA to infected astrocytes to ensure the transfect efficiency of siRNA. Then the transfected cells were treated with medium or IL-17A for the subsequent experiments.

### Isolation, Culture, and Identification of Oligodendrocytes Lineage Cells

Fresh neonatal mice (1-3 days) brain tissues were obtained as the above method. The cortex was separated and cut into small pieces with ophthalmic scissors, and then moved into the centrifugal tube together with HBSS solution. The tissue was gently blown several times with the head of a gun until obvious tissue could not be seen by the naked eye. The tissue suspension was collected and centrifuged at 1000rpm for 5min. The supernatant was removed and the cells were washed repeatedly. Cell precipitation was blown again with neural stem cells (NSCs) growth culture medium (DMEM/F12 supplemented with 1% B27 and 20ng/ml bFGF), and the cell suspension was transferred to a culture flask for suspension culture at 37°C in an incubator of 95% air and 5% CO2. After NSCs formed the neurosphere, they were dissociated and transferred into oligodendrocyte progenitor cells (OPCs) induction medium (DMEM/F12 supplemented with 1% B27, 20ng/ml bFGF, 30ng/ml triiodothyronine, and 10 ng/ml PDGF-AA). We can use differential digestion and differential adhesion method to purify OPCs. The OPCs were cultured in the mature differentiation medium (DMEM/F12 supplemented with 1% B27, 1%N2, 20 ng/mL PDGF-AA, and 20 ng/mL IGF1, 40ng/ml T3, and 5% FBS) until the cells were fully mature and formed a “spiderweb-like” structure of oligodendrocyte and were identified by specific cell markers.

### RNA Isolation and Real-Time PCR

Total RNA was extracted from the brain using a trizol reagent (TaKaRa) according to the instructions. mRNA was then reversely transcribed using the Prime Script RT reagent kit (TaKaRa) for cDNA synthesis. Then we used cDNA to perform real-time quantitative PCR using the SYBR Premix Ex Taq kit (TaKaRa). The 2-ΔΔCt method was used to assess the relative expression level of mRNA. mRNA levels were measured using the following specific primer sequences: C3d, 5’-CGTGGCCAAGCTAAGCATCA-3’ and 3’-GGCCTCCATTGTCTTGGTGG -5’;S100α10, 5’-TTGCAGGCGACAAAGACCAC-3’, 3’-CACTTTGCCATCTCGGCACT-5’; SOCS3, 5’-CTGGTACTGAGCCGACCTCT -3’ and 3’-GGCAGCTGGGTCACTTTCTC -5’;STAT3, 5’-CATTGACCTGCCGATGTCCC -3’ and 3’-GAGCGACTCAAACTGCCCTC -5’. Expression of the gene of interest was normalized to that of the housekeeping gene GAPDH (reduced glyceraldehyde-phosphate dehydrogenase).

### High Throughput Sequencing and mRNA Library Construction

Our research group set RNA sequencing data library of mice brain infected with *A. cantonensis* (26). Mice were separately sacrificed at 2, 7, 14, 21 days post-infection with *A. cantonensis*, and the mice brains were immediately isolated to extract total RNAs. The same number of healthy mice samples were used as control. Briefly, the total RNAs contained were extracted using the RNeasy^®^ Mini Kit (Qiagen, Germany) and reversed transcription to cDNA to prepare the Illumina/Solexa sequencing library. And then we used Illumina Genome Analyzer IIx to perform the sequencing process. The expression profiles of the SOCS family were shown in a heatmap.

### Histology and Immunofluorescence

Mice were anesthetized with isoflurane and perfused transcardially with ice-cold PBS followed by 4% paraformaldehyde (PFA). After fixing with PFA and 30% sucrose water, brain samples were embedded in optimal cutting temperature compound (OCT) and cut into10 μm slides at -20°C. Next, slides were permeabilized with 0.3% Triton X-100 and blocked with 2% bovine serum albumin (BSA) for 1 h at room temperature. Removing blocking buffer and incubating slides with the primary antibody in 1% BSA at 4°C overnight. Slides were washed in PBS three times and incubated with fluorescein isothiocyanate-labeled secondary antibody, which diluted 1:500 in 1% BSA at 37°C for 1 h. DAPI was then applied for 5 mins to stain the nucleus. Slides were washed again in PBS and observed under a fluorescence microscope. The antibodies used to detect the cells were as follows: anti-GFAP (CST, 3670), anti-S100β (Abcam, ab52642), anti-SOCS3 (Abcam, ab16030), anti-C3d (RD system, AF2655), anti-S100a10 (RD system, AF2377), and anti-MBP (Abcam, ab40390), anti-oligo2 (Sigma-Aldrich, AB9610). Staining samples without the primary antibody were used as negative controls.

### Western Blotting

Brain tissue was lysed in extraction buffer (20 mM HEPES [pH 7.4], 2 mM EDTA, 50 mM glycerophosphate, 1 mM dithiothreitol, 1 mM Na3VO4, 1% Triton X-100, and 10% glycerol) on ice. The lysates were centrifuged at 1000 rpm for 10 mins. Supernatants were collected and their protein concentrations were determined using a bicinchoninic acid protein assay (BCA assay). Proteins were heated with sample buffer, separated in 12% sodium dodecyl sulfate-polyacrylamide gels by electrophoresis, and electroblotted onto a nitrocellulose membrane. Transferred blots were incubated with a blocking agent (5% non-fat milk in Tris-buffered saline). Anti-SOCS3, anti-STAT3(CST, 0139), anti- phosphor-STAT3(CST, 9145), and their secondary antibody blots were developed using the enhanced chemifluorescence detection kit according to the manufacturer’s instructions. The same blots were subsequently stripped and reblotted with an antibody of β-tubulin to verify equal protein loading. Graphs of blots were obtained in the linear range of detection and protein levels were quantified using ImageJ software (NIH).

### Quantification and Statistical Analysis

The random number generator function in Microsoft Excel was used to randomly allocate experiment mice. Positive cell counts and lesion pixel area quantification for Western Blotting were performed using ImageJ. GraphPad Prism 8.2 was used to statistically compare the data on real-time quantitative PCR, Western Blot, and immunofluorescence graphs among different groups. Data are expressed as the mean ± SEM and were analyzed using a two-tailed t-test or one-way ANOVA and Tukey’s test, as appropriate. p < 0.05 was considered statistically significant.

## Results

### *A. cantonensis* Infection Causes Activation of Type A1 and A2 Astrocytes in the Corpus Callosum

**Figure 1:**
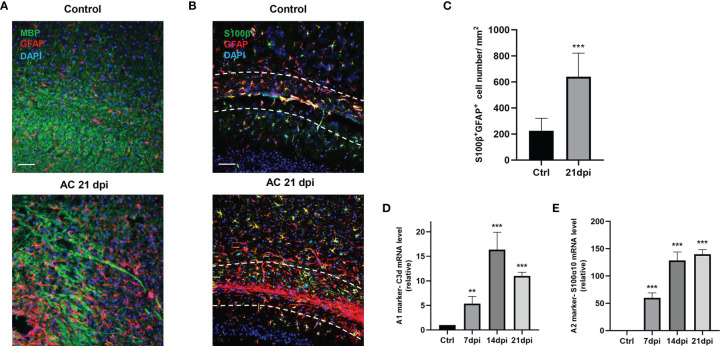
*A. cantonensis* infection caused astrocytes activation. **(A)** Representative sections showing that immunostaining distribution MBP and GFAP in control (Ctrl) group and 21 dpi group. Scale bar = 75 μm. **(B)** Representative images of S100β and GFAP staining of control and 21 dpi groups. Scale bar = 75 μm. **(C)** Quantification of GFAP^+^, S100β^+^ cells. **(D, E)** mRNA expression of C3d and S100α10 of the brain at 7, 14, and 21 dpi. **P < 0.01, ***P < 0.001. Data were analyzed by one-way ANOVA and followed by Tukey’s *post hoc* analysis. Data in each statistical graph are presented as the mean ± SEM.

*A. cantonensis* infection is a typical parasitic infection that causes serious brain injury. In the late stage of infection, almost all larvae of *A. cantonensis* cross the BBB and parasitize the brain (27). As we have mentioned, demyelination is a typical pathological change in *A. cantonensis* infection and its severity is proportional to motor dysfunction in infected mice. Similar to our previous study, staining for myelin basic protein (MBP) showed apparent demyelination, especially at 21 days post-infection (dpi) (**Figure 1A**). In the present study, we attempted to identify the role of glial cells in this process. We detected different drastic astrocyte activation around the corpus callosum by staining the astrocyte markers, GFAP and S100β (**Figures 1B, C**). Importantly, astrocytes exhibited prolonged cell processes and displayed a hypertrophic cellular body, which is a typical phenotype for reactive cells.

**Figure 2:**
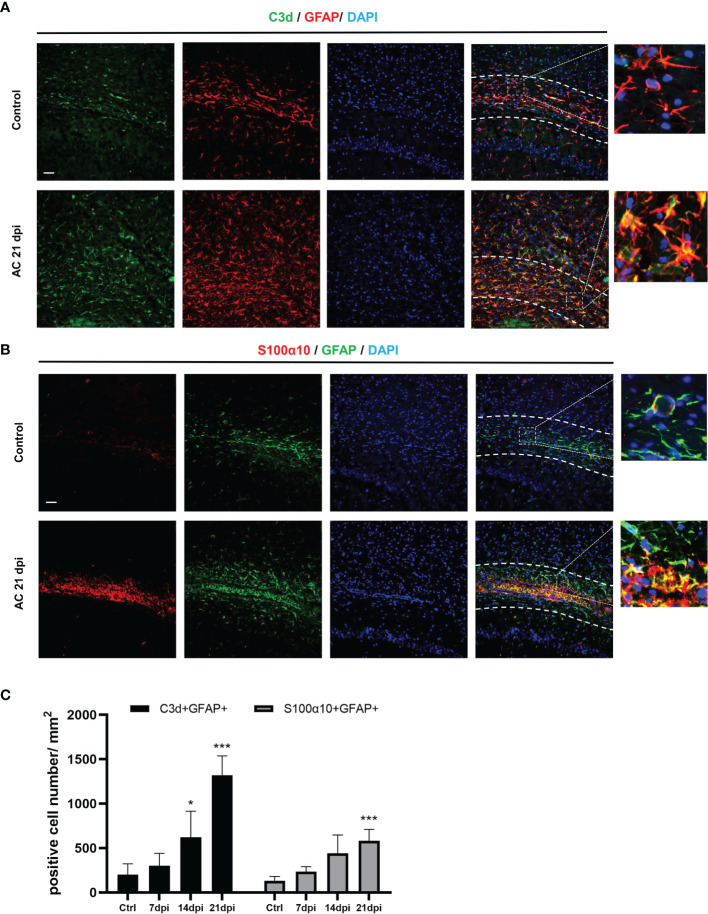
*A. cantonensis* infection caused activation of A1 and A2 astrocytes in the corpus callosum. **(A, B)** Representative sections showing that distribution of GFAP^+^, C3d^+^ and GFAP^+^, S100α10^+^ cells in the corpus callosum. **(C)** Quantification of GFAP^+^, C3d^+^ and GFAP^+^, S100α10^+^ cells at 7, 14, and 21 dpi. Scale bar = 50 μm. n = 4-5 animals/group, *P < 0.05, ***P < 0.001. Data were analyzed by one-way ANOVA and followed by Tukey’s *post hoc* analysis. Data in each statistical graph are presented as the mean ± SEM.

To determine the type of activated astrocytes, we assessed the levels of the astrocyte activated marker genes, C3d and S100α10, which represent A1 astrocytes and A2 astrocytes, respectively. Both genes expression increased during infection (**Figures 1D, E**). Immunofluorescence staining results also showed that the number of astrocyte positive cells (C3d^+^ GFAP^+^, S100α10^+^ GFAP^+^) increased significantly at 14 dpi (**Figures 2A, B**). These results indicated that both A1 and A2 astrocytes were highly activated after *A. cantonensis* infection and that the activation of astrocytes was mainly concentrated in the corpus callosum region.

### IL-17A Mediates the Activation of A1 Astrocytes During *A. cantonensis* Infection

**Figure 3:**
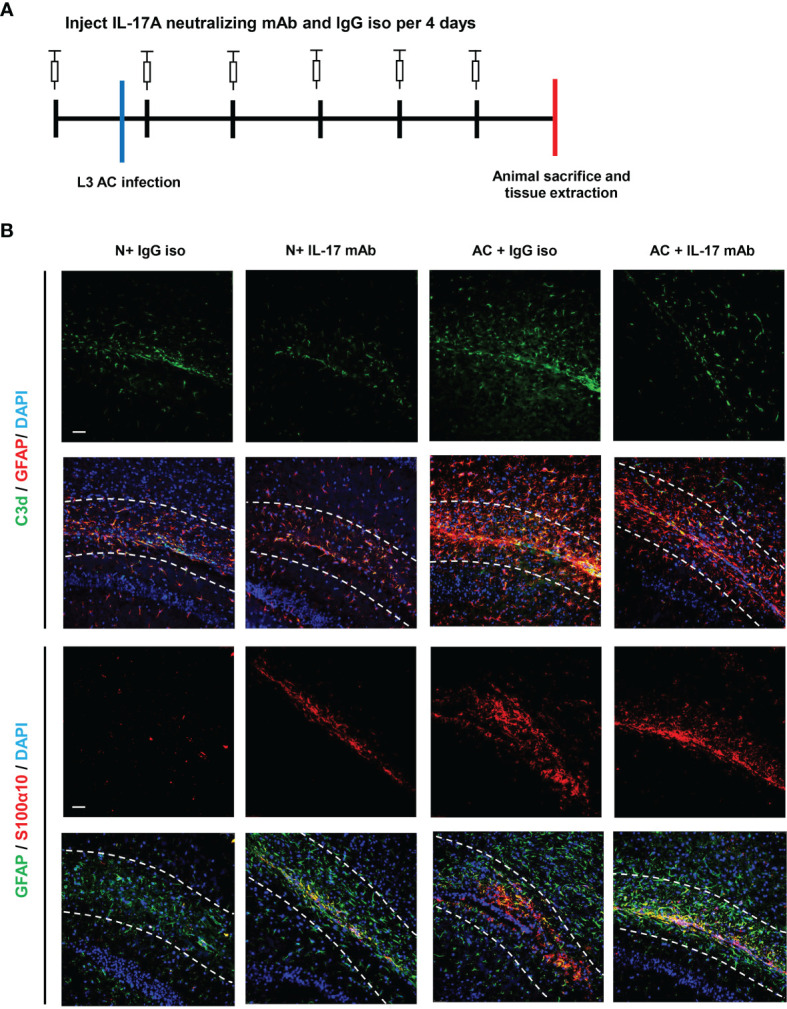
IL-17A mediated the activation of A1 astrocytes during *A. cantonensis* infection. **(A)** Flow chart of IL-17A mAb and IgG isotype administration to mice infected with *A. cantonensis*. **(B)** Representative sections of GFAP^+^, C3d^+^, and S100α10^+^ immunostaining from mice treated with IL-17A neutralizing antibody and IgG1 isotype antibody. Scale bar = 50 μm. n = 4-5 animals/group. Data in each statistical graph are presented as the mean ± SEM.

**Figure 4:**
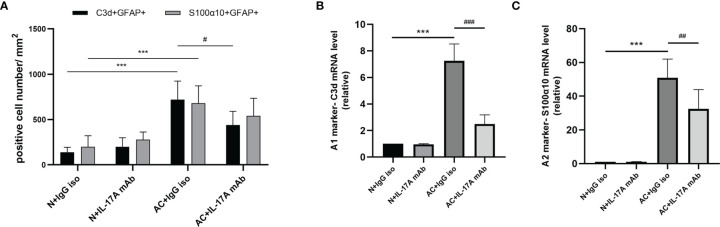
IL-17A mediated the activation of A1 astrocytes during *A. cantonensis* infection. **(A)** Quantification of GFAP^+^, C3d^+,^ and GFAP^+^, and S100α10^+^ in different groups. **(B, C)** mRNA expression of C3d and S100α10 of the brain of four groups. n = 4-5 animals/group, ***P < 0.001. Data were analyzed by one-way ANOVA and followed by Tukey’s *post hoc* analysis. Data in each statistical graph are presented as the mean ± SEM. ^#^P < 0.05, ^##^P < 0.01, ^###^P < 0,001.

We previously observed that IL-17A levels increased after *A. cantonensis* infection and injection of neutralizing antibodies successfully decreased IL-17A to a low level. It has been proven that injecting IL-17A neutralizing antibodies can relieve the degree of demyelination (25). Next, we wanted to determine whether astrocytes mediated this process (**Figure 3A**). Immunofluorescence staining results showed that the expression of GFAP in brain tissues decreased after IL-17A was neutralized and C3d expression was also reduced by a statistically significant amount compared with the control group, while there was no significant difference in the expression of S100α10 (**Figures 3B**, **4A, C**). qPCR analysis of these genes showed similar results (**Figures 4B, C**). This suggested that IL-17A mainly mediated the activation of A1 astrocytes during *A. cantonensis* infection.

**Figure 5:**
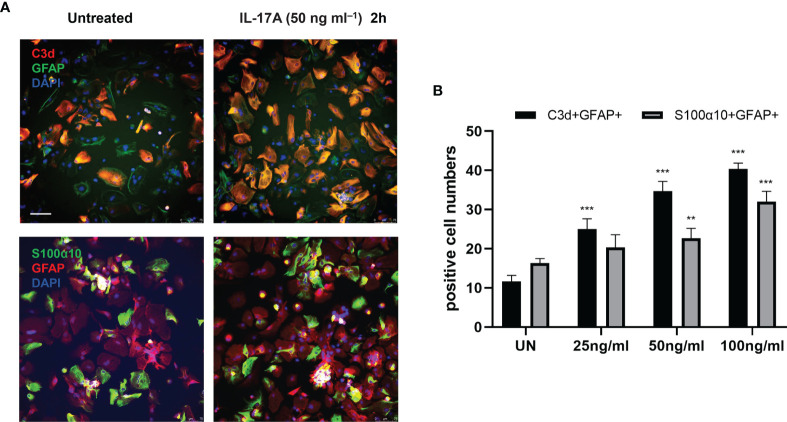
IL-17A activated astrocytes *in vitro*. **(A)** Representative images display the expression of GFAP, C3d, and S100α10 in the presence of different concentration of IL-17A (UN=untreated). Scale bar = 75 μm. **(B)** Quantification of GFAP^+^, C3d^+^, and GFAP^+^ S100α10 cells of each treated group. n = 3/group, **P < 0.01, ***P < 0.001. Data were analyzed by one-way ANOVA and followed by Tukey’s *post hoc* analysis. Data in each statistical graph are presented as the mean ± SEM.

Next, we explored whether IL-17A has the capacity to influence astrocytes *in vitro* by stimulating primary astrocytes with different concentrations of IL-17A. The results showed 50 ng/mL was the optimal stimulating concentration of IL-17A. Following treatment with IL-17A, astrocytes were activated and cell expression of C3d and S100α10 increased, with a significant increase especially in C3d^+^ GFAP^+^ cell numbers (**Figures 5A, B**).

### IL-17A Regulates SOCS3 Expression in Astrocytes Through the IL-17RA/STAT3/SOCS3 Pathway

**Figure 6:**
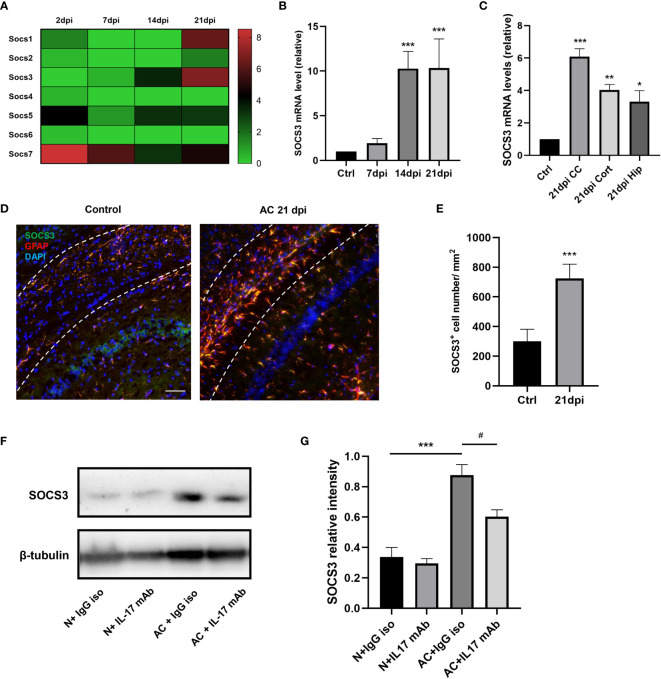
IL-17A regulated SOCS3 expression in astrocytes during *A. cantonensis* infection. **(A, B)** Results of gene enrichment analysis was processed through comparing SOCS family mRNA levels and qPCR results of SOCS3 in infected mice brains at 2, 7, 14, and 21 dpi. **(C)** qPCR assay was performed to assess SOCS3 gene levels of different brain regions at 21 dpi (CC = corpus callosum, Cort = cortex, Hip = hippocampus). **(D)** Representative images of SOCS3 and GFAP staining of control and 21 dpi groups. Scale bar = 75 μm. **(E)** Quantification of GFAP^+^, SOCS3^+^ cells at 7, 14, and 21 dpi. **(F)** Western Blotting results showed SOCS3 protein levels of individual treated groups. **(G)** Relative densitometric analysis of Western Blotting is represented, as normalized to β-actin. n = 5 animals/group, *P < 0.05, **P < 0.01, ***P < 0.001, ^#^P < 0.05. Data were analyzed by one-way ANOVA and followed by Tukey’s *post hoc* analysis. Data in each statistical graph are presented as the mean ± SEM.

The above results suggest that IL-17A is involved in the activation of A1 astrocytes during *A. cantonensis* infection. Through our previous gene sequencing and qPCR verification, it was proven that the mRNA level of the SOCS3 gene in astrocytes increased significantly at 14dpi and this high expression level persisted until the late stage of infection (**Figures 6A–C**). SOCS3 protein is considered to inhibit axonal regeneration, therefore we hypothesized that IL-17A induced high expression levels of SOCS3 in astrocytes, resulting in sustained myelin injury after *A. cantonensis* infection. Immunofluorescence staining was used to co-label SOCS3 and GFAP proteins and it was found that SOCS3 was highly expressed in astrocytes but not in other cells after infection (**Figures 6D, E**). Western blotting was used to detect changes in SOCS3 protein content after injection of the neutralizing antibody. The results showed that the expression level of IL-17A was positively correlated with SOCS3 and that the SOCS3 expression level in mice of the IL-17 neutralizing antibody group was lower than that of the control group, with the difference being statistically significant (**Figures 6F, G**).

**Figure 7:**
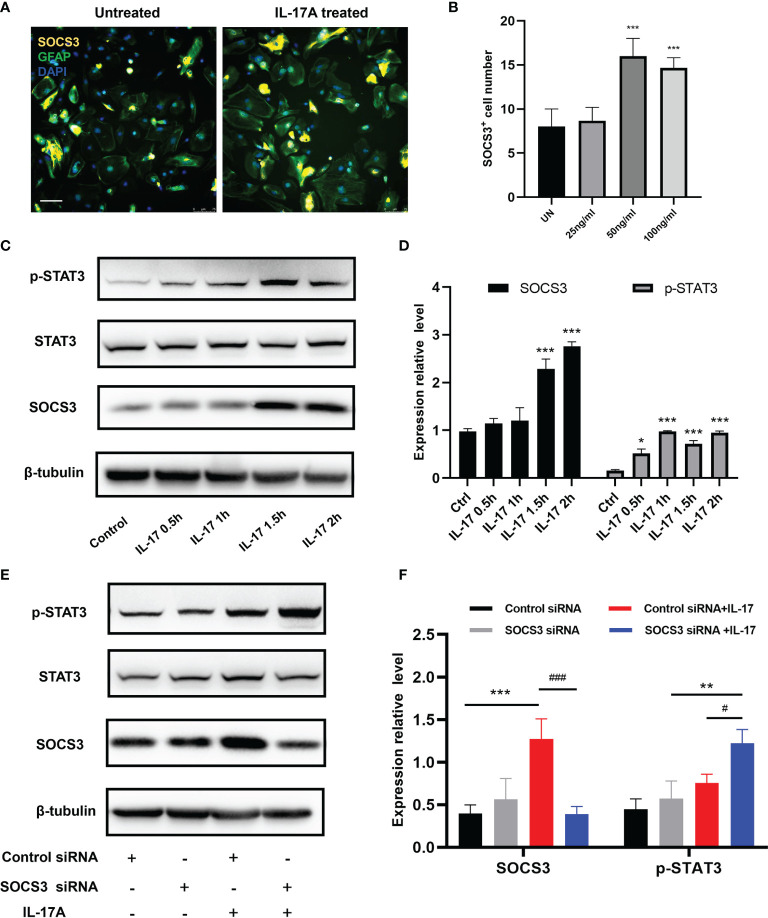
IL-17A regulated SOCS3 expression in astrocytes through the IL-17RA/STAT3/SOCS3 pathway. **(A)** Representative images display the expression of SOCS3, GFAP in the presence of a different concentration of IL-17A. Scale bar = 75 μm. **(B)** Quantification of C3d^+^, SOCS3^+^ cells in different treated groups. **(C)** Representative immunoblotting of SOCS3, STAT3, p-STAT3, and β-tubulin in several different stimulating time groups (0.5 h, 1 h, 1.5 h, 2 h). **(D)** Relative densitometric analysis of Western Blotting is represented, SOCS3 is normalized to β-tubulin, p-STAT3 is normalized to STAT3. **(E)** Representative Western Blotting images display the expression of SOCS3, STAT3, p-STAT3, and β-tubulin in the presence or absence of IL-17A and SOCS3 siRNA. **(F)** Relative densitometric analysis of Western Blotting in each treatment group is represented, SOCS3 is normalized to β-tubulin, p-STAT3 is normalized to STAT3. n = 3, ***P < 0.001, ^#^P < 0.05, ^###^P < 0,001. Data were analyzed by one-way ANOVA and followed by Tukey’s *post hoc* analysis. Data in each statistical graph are presented as the mean ± SEM.

The traditional pathway involving SOCS3 is the JAK/STAT pathway, therefore we further investigated the expression levels of the STAT family. We processed the IL-17A stimulation experiment in astrocytes to ascertain the expression patterns of SOCS3 and STAT3. SOCS3 and p-STAT3 expression levels were strongly increased along with the stimulation time extension (**Figures 7A–D**). To examine whether SOCS3 participates in the activation of astrocytes, a specific knockdown experiment of SOCS3 with siRNA was performed in primary astrocytes and astrocytes were then treated with an appropriate concentration of IL-17. The p-STAT3 protein was no longer inhibited by SOCS3, which indicated that IL-17A activated astrocytes through IL-17RA *via* the STAT3/SOCS3 pathway (**Figures 7E, F**). In conclusion, IL-17A promoted tyrosine phosphorylation of STAT3 and p-STAT3 induced the expression of SOCS3. In turn, phosphorylation of STAT3 was inhibited by SOCS3 in astrocytes.

### High Expression of SOCS3 in A1 Astrocytes During *A. cantonensis* Infection Results in Myelin Injury

**Figure 8:**
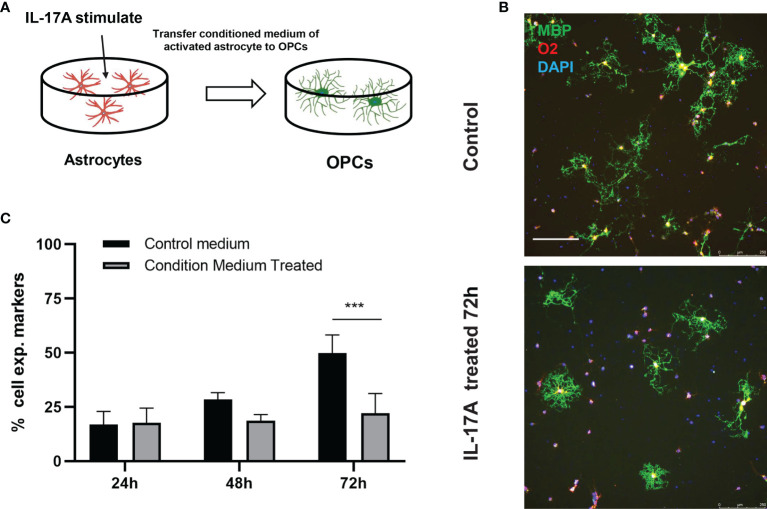
IL-17A activation of astrocytes hindered oligodendrocytes formation. **(A)** Schematic diagram showing the collection of astrocyte medium after treatment with IL-17A which was then mixed with OPCs mature medium in 1:1 ratio to form conditioned medium and applying conditioned medium to OPCs. **(B)** Representative images of oligodendrocytes staining images of control or conditioned medium treated groups. Scale bar = 250 μm. **(C)** Percentage of MBP^+^ cells to total cell numbers. n = 3, ^##^P < 0.01, ***P < 0.001, one-way ANOVA and Tukey’s test. Data in each statistical graph are presented as the mean ± SEM.

**Figure 9:**
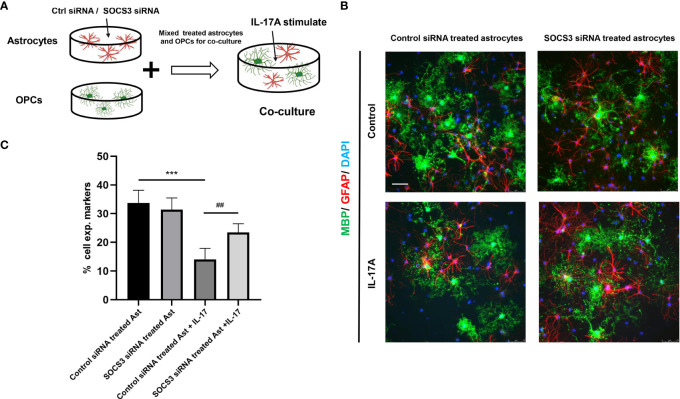
High expression of SOCS3 in activated A1 astrocytes inhibited oligodendrocyte formation. **(A)** Schematic diagram showing the collection of astrocytes treated with control siRNA or SOCS3 siRNA, followed by culturing astrocytes with OPCs in the presence or the absence of IL-17A. **(B)** Representative images of OPCs cultured with astrocytes treated with control siRNA or SOCS3 siRNA for 72 h, which were immunostained for MBP and GFAP. Scale bar = 75 μm. **(C)** Percentage of MBP^+^ cells to total cell numbers. n = 3, *P < 0.05, **P < 0.01, ***P < 0.001, one-way ANOVA and Tukey’s test. Data in each statistical graph are presented as the mean ± SEM.

The myelin sheath is mainly formed by the myelination of oligodendrocytes in the central nervous system. Direct damage to oligodendrocytes or inhibition of the differentiation process of oligodendrocyte progenitor cells (OPCs) to oligodendrocytes is potential causes of demyelination. Some studies have indicated that A1 astrocytes act as harmful cells that inhibit OPC differentiation (28). To illustrate the relationship between IL-17A, astrocytes, and oligodendrocytes, we used indirect stimulation and cell co-culture methods. The conditioned medium from the primary astrocytes treated with IL-17A was collected and applied to primary cells for 24, 48, 72 h to verify the effects of activated astrocytes (**Figure 8A**). Oligo2 (O2) and MBP, as two typical oligodendrocyte markers, were used to stain the mature oligodendrocytes as a previous study (29). The results suggested that the oligodendrocyte number decreased with stimulating time processing (**Figures 8B, C**). To further investigate the damaging effects of activated astrocytes induced by IL-17A on oligodendrocytes, we cultured astrocytes on top of OPCs and maintained the co-culture in the presence or absence of IL-17A for 72 h. Astrocytes were previously treated with control siRNA or SOCS3 siRNA (**Figure 9A**). We then used a similar method to assess the number of MBP^+^ oligodendrocytes out of the total cell count. Compared with the control group, astrocytes interfered with by si-SOCS3 exhibited weakened effects on OPCs after IL-17A stimulation (**Figures 9B, C**). Hence, the damaging effect of IL-17A activated astrocytes on oligodendrocytes can be blocked through the interference of the IL-17RA/STAT3/SOCS3 pathway. Therefore, we speculated that myelin damage during *A. cantonensis* infection is partially attributed to IL-17A induced SOCS3 activation in astrocytes.

## Discussion

Abnormal myelin development or damage can result in abnormal impulse transmission. Myelination is also important for cognitive function and studies have shown that myelin is involved in the development of motor learning and social interaction (30, 31). These highlight the importance of exploring the mechanism of demyelination. Our last published research showed the role of microglia in *A. cantonensis* infection, some cytokines secreted by microglia had been proved to promote the differentiation of astrocytes, such as C1q, IL-11α, and TNF (14), we subsequently found obvious astrocytes activation during this process. This indicates that the interaction between cells is important in the progression of the disease.

IL-17 is a typical pro-inflammatory factor that can induce inflammatory cell infiltration and tissue destruction, especially in autoimmune diseases such as type 1 diabetes (32, 33). At present, different studies have shown varied views on the relationship between IL-17A and demyelinating diseases. Multiple sclerosis is a representative central nervous system autoimmune disease and its animal model is EAE. It has been reported that EAE modeling in IL-17^-/-^ mice lead to MOG-specific T cell sensitization, resulting in significantly reduced severity of disease damage (34). Th17 cells can interact synergistically with γδ T cells by producing IL-17 and IL-23 to accelerate disease progression in EAE models (35, 36). IL-17 is also involved in fighting off parasitic infections. Studies have shown that the secretion of IL-17 is promoted by chil3 and IL-17 and this has been shown to recruit neutrophils to fight against *Nippostrongylus brasiliensis* infection (37).

Our results showed that the increase of IL-17A induced by *A. cantonensis* infection not only caused myelin sheath injury but also activated astrocytes, with both A1 and A2 astrocytes being activated to varying degrees. In our infection model, the degree of activation of astrocytes positively correlated with the expression of IL-17A, and astrocytes were activated earlier than microglia. Therefore, we detected the activation status of astrocytes after IL-17A neutralization and the results showed that the activation of A1 astrocytes was significantly inhibited, similar to the results of *in vitro* experiments. Previous studies have shown that A1 astrocytes mainly promote inflammation, whereas A2 astrocytes inhibit inflammation (14). The changes in IL-17A, demyelination injury, and A1 astrocytes were consistent, so we inferred that the activation of A1 astrocytes induced by IL-17 might be related to demyelination injury. It has been shown that in EAE models, the activation of astrocytes is affected by IL-17 secreted by Th17 cells and this activation process is mainly mediated by the Act1 gene. Act1-specific deletion of astrocytes can interrupt the IL-17-induced cascade inflammatory response and thus affects the progression of EAE (38). In addition, astrocytes express the ubiquitin-modified enzyme A20, which inhibits the expression of the NF-κB pathway. Conditional knockdown of the A20 gene in astrocytes results in an uninhibited NF-κB pathway that exacerbates EAE progression (39). In the *in vitro* experiments, we incubated oligodendrocytes in the medium transferred from IL-17 stimulated astrocytes. The axons of oligodendrocytes were damaged and cell number decreased, which suggested IL-17A activated A1 astrocytes had an inhibiting effect on the growth of the oligodendrocytes.

Abnormal SOCS3 expression was also detected after *A. cantonensis* infection and IL-17A stimulation led to upregulation of SOCS3 expression in astrocytes *in vitro*. This process was short-lived and the highest level of SOCS3 expression was approximately 2h after IL-17A was applied, thereafter SOCS3 expression declined over time. A previous study demonstrated that natural killer cell activity can be inhibited by IL-17A through SOCS3, *w*hich can interfere with tumorigenesis and viral infection (40). SOCS3 plays an important role in various nervous systems. Specific knockout of SOCS3 in neurons leads to increased leptin sensitivity in the hypothalamus region, thus inhibiting appetite and reducing body weight (41). SOCS3 is an inhibitor of STAT3 and the error of the SOCS3 regulation program on STAT3 leads to abnormal STAT3 expression in the brain tissue of glioma patients, which is due to phosphorylated STAT3 promoting the expression of many tumorigenesis genes (42). SOCS3 has been proven to be related to the axon growth process and SOCS3 knockout in mice can accelerate the recovery rate of axon-damaged nerve fibers (43). Previous studies have shown that *A. cantonensis* infection can also cause an increase in IL-6 and IFN-γ levels (44) and these two proven factors can also promote SOCS3 expression through the JAK/STAT pathway (22). Moreover, IL-17A can increase SOCS3 protein levels through upregulation of IL-6 expression, which then activates astrocytes (45). It has been shown *in vitro* that IFN-β can promote the expression of SOCS1 and SOCS3 in astrocytes, wherein STAT1α is the key protein of SOCS1 and STAT3 is the key protein of SOCS3, and the co-expression of SOCS1 and SOCS3 affects the secretion of chemokines in astrocytes (46).

Common demyelination disease including multiple sclerosis, optical neuromyelitis, Guillain-Barre Syndrome, etc. Many evidences indicated IL-17 play an important role in these diseases (47). Our studies first proposed that IL-17A-triggered SOCS3 mediated astrocyte activation involved *A. cantonensis* caused demyelination. However, more detailed mechanisms about how SOCS3 regulate astrocyte activation to influence myelin damage process should be studied. In the future research, we plan to specifically interfere SOCS3 gene in mice astrocyte to observe its functions in *A. cantonensis* infection induced demyelination. In addition, the effects of IL-17A and SOCS3 in typical demyelination animal model should be explored to provide more thoughts for intervening myelin damage.

## Conclusion

Our present study showed both A1 and A2 astrocytes apparent activation after *A. cantonensis* infection and promoted the demyelination of corpus callosum. Furthermore, we firstly proposed that SOCS3 not only mediated A1 astrocytes activation, but also accelerated demyelinating injury in an *A. cantonensis-*infected animal model. Therefore, the reintroduction of a specific gene SOCS3, in astrocytes could be investigated as a potential method to slow down the progression of demyelination.

## Data Availability Statement

The multiple infectious stages of the *A. cantonensis* infected mice brain tissue RNAseq reads have been deposited at NCBI in the SRA (BioProject number: PRJNA803318). The original contributions presented in the study are included in the article. Further inquiries can be directed to the corresponding authors.

## Ethics Statement

The animal study was reviewed and approved by Institutional Animal Care and Use Committee of Sun Yat-Sen University.

## Author Contributions

ZZ, TL, and ZL carried out the experiments, ZZ, QD, ZM, and QD performed the statistical analyses. ZZ drafted the manuscript. FK, YL, and YF conceived and coordinated the study. All authors read and approved the final manuscript.

## Funding

This work was supported by the National Natural Science Foundation of China [grant numbers 81401688, 8180051466, 81772438, 81974357]; the Guangzhou Municipal Science and Technology Program [grant number 201803010083]; Fundamental Research Funds for the Central Universities, SCUT (grant number 2018MS81); South China University of Technology Scientific Research Funding (grant number D6172910).

## Conflict of Interest

The authors declare that the research was conducted in the absence of any commercial or financial relationships that could be construed as a potential conflict of interest.

## Publisher’s Note

All claims expressed in this article are solely those of the authors and do not necessarily represent those of their affiliated organizations, or those of the publisher, the editors and the reviewers. Any product that may be evaluated in this article, or claim that may be made by its manufacturer, is not guaranteed or endorsed by the publisher.
